# The AcasR-FadD9 regulatory axis modulates iron homeostasis and susceptibility to the anti-mycobacterial compound ACA

**DOI:** 10.3389/ftubr.2026.1830528

**Published:** 2026-06-30

**Authors:** Feng Huang, Zixin Wang, Dong Jiang, Shengyi Li, Zheng-Guo He, Hua Zhang

**Affiliations:** 1 National Key Laboratory of Agricultural Microbiology, College of Life Science and Technology, Huazhong Agricultural University, Wuhan, China; 2 State Key Laboratory of Virology, Taikang Center for Life and Medical Sciences, TaiKang Medical School (School of Basic Medical Sciences), Wuhan University, Wuhan, China; 3 College of Life Science and Technology, Guangxi University, Nanning, China

**Keywords:** ACA sensitivity, AcasR, FadD9, iron homeostasis, mycobacterium

## Abstract

Mycobacterial drug resistance leads to prolonged treatment, increased mortality, and increased healthcare costs. In this study, 3-azidothiophene-2-carboxylic acid (ACA), a dual-targeting inhibitor of the cell assembly proteins CpsA1 and CpsA2, exerted its activity through an iron-dependent pathway. The screening of drug-resistant mutants revealed that a frameshift mutation in the transcriptional regulator *acasR* (BCG_0932/Rv0880) conferred ACA resistance. Conversely, *acasR* overexpression increased bacterial susceptibility. Furthermore, AcasR specifically bound to and repressed the promoter of *fadD9* (Rv2590), which was its sole functionally validated target under the tested conditions. Crucially, *fadD9* overexpression phenocopied ACA resistance, whereas its deletion heightened susceptibility. Integrated omics and iron assays revealed that ACA induced intracellular iron starvation, a condition exacerbated by *fadD9* deletion. These findings reveal a novel resistance pathway in which AcasR modulates mycobacterial drug susceptibility by repressing *fadD9*, which in turn affects siderophore-mediated iron homeostasis.

## 1 Introduction

Tuberculosis (TB), an infectious disease caused by *Mycobacterium tuberculosis*, remains a major global public health problem. In total, 8.2 million new cases and 1.25 million TB deaths were reported in 2023. Thus, TB is likely to become the world's leading cause of death from a single infectious agent (replacing COVID-19). Drug-resistant TB (MDR/RR-TB) remains a major threat. Of the 400,000 cases estimated worldwide in 2023, only 44% were diagnosed and treated (30).

3-azidothiophene-2-carboxylic acid (ACA) is a newly discovered anti-TB small-molecule compound that simultaneously targets the cell assembly proteins CpsA1 and CpsA2 (13). As cell core assembly represents a novel antimicrobial target distinct from all current TB drugs, its combination with existing therapies could provide synergy while reducing the emergence of resistance. CpsA1 and CpsA2 exhibit partially redundant functions, consequently allowing bacteria to rely on either gene for essential cellular processes (10). For example, single mutants (Δ*cpsA1* or Δ*cpsA2*) can be obtained, whereas the double deletion (Δ*cpsA1* Δ*cpsA2*) is lethal (10). This indicates that concurrent mutations in both genes are required for mycobacteria to completely escape drug inhibition. However, the likelihood of this event is extremely low owing to the multiplicative nature of mutation rates. Even if a bacterium acquires a mutation in one gene (e.g., c*psA2*), the remaining functional copy (*cpsA1*) would be susceptible to drug targeting. This may explain why ACA can inhibit the growth of the ACA-resistant mutant at 32 μg/mL (13).

MarR regulators are proteins belonging to multiple antibiotic resistance regulator (MarR) family. They regulate the activity of genes involved in stress responses, virulence, fatty acid metabolism, and antibiotic resistance (11, 15). The *M. tuberculosis* genome encodes at least nine putative MarR family transcriptional regulators, of which Rv0678 is particularly well-characterized (2, 9, 22). This regulator functions as a repressor of the mmpS5-mmpL5 efflux pump operon, which mediates the extrusion of bedaquiline (BDQ) and clofazimine (2, 18). Clinically significant resistance to these drugs is associated with specific *rv0678* mutations that impair their DNA-binding capacity at the mmpS5-mmpL5 promoter region (14).

Additional regulatory mechanisms affecting BDQ susceptibility have been identified through studies of other transcriptional regulators. Peterson et al. (21) demonstrated that Rv0880 (a MarR homolog) and Rv0324 (an ArsR family protein) contributed to intrinsic BDQ tolerance. Genetic deletion of either *rv0324* or *rv0880* markedly increased BDQ sensitivity while maintaining normal susceptibility to other antimycobacterial agents with distinct mechanisms of action (17).

In this study, we generated an ACA-resistant mutant strain with a mutation in BCG_0932/Rv0880 (designated AcasR, ACA-sensitivity repressor). AcasR modulated strain sensitivity to ACA by regulating the expression of *fadD9*. Deletion of this gene enhanced mycobacterial susceptibility to ACA. One possible mechanism is that ACA treatment induces iron starvation in mycobacteria, whereas f*adD9* deletion further downregulates siderophore biosynthesis, thereby rendering the *fadD9*-deficient strain more sensitive to ACA.

## 2 Materials and methods

### 2.1 Strains, plasmids, enzymes, and reagents

*Escherichia coli* BL21 (*λ* DE3) and pET28a were purchased from Novagen (Darmstadt, Germany) and used for protein expression. Restriction enzymes, T4 ligase, modification enzymes, DNA polymerase, and dNTPs were purchased from TaKaRa Bio (Shiga, Japan). Polymerase chain reaction (PCR) primers were synthesized by Invitrogen (Carlsbad, CA, USA). Ni^2+^-nitrilotriacetate (Ni-NTA) agarose was purchased from Qiagen (Hilden, Germany). 7H9 and 7H10 broths were purchased from Becton Biosciences (Franklin Lakes, NJ, USA). Antibodies were obtained from the Wuhan Laboratory Animal Center of CAS (Wuhan, China). ACA was purchased from Specs (Delft, Netherlands). All mycobacterial strains were grown in 7H9 medium (supplemented with 10% OADC, 0.05% Tween-80, and 0.2% glycerol) or 7H10 medium (supplemented with 10% OADC and 0.5% glycerol) at 37 °C and shaken at 160 rpm.

### 2.2 Cloning, expression, and purification of recombinant proteins

Genes were amplified through PCR using specific primer pairs (Supplementary Table 1) and templates from the genomic DNA of *M. tuberculosis* H37Rv. The amplified DNA fragments were cloned into the modified pET28a expression vectors and pMind or pMV261 overexpression vectors to produce recombinant plasmids (Supplementary Table 2). *E. coli* BL21 cells were used to express the recombinant proteins. Recombinant *E. coli* BL21 cells were grown in 1 L of Luria broth (LB) medium to an OD_600_ of 0.6. AcasR expression was induced by adding 1 mM isopropyl *β*-D-1-thiogalactopyranoside at 16 °C for 18 h. Harvested cells were resuspended and sonicated in ice-cold binding buffer (20 mM Tris-HCl, pH 8.0; 500 mM NaCl, and 5 mM imidazole). The lysate was centrifuged at 10,000  × *g* for 30 min and the supernatant was loaded onto an affinity column (Ni-NTA agarose affinity matrix). The column-bound protein was washed with wash buffer (20 mM Tris-HCl, pH 8.0; 500 mM NaCl, and 50 mM imidazole). The elution was dialyzed for 2 h and stored in buffer (20 mM Tris-HCl, pH 7.5; 100 mM NaCl, and 10% glycerol) at −80 °C. Protein concentration was determined using the Bradford method (5).

### 2.3 Screening and characterization of ACA-resistant mutants

To screen for transcription factors that regulate mycobacterial susceptibility to ACA, we used a pre-constructed *M. tuberculosis* transcription factor overexpression library to identify ACA-resistant transformants. 7H10 agar plates containing 4 × and 8 × the MIC of ACA were prepared to select resistant transformants. Log phase *Mycobacterium bovis* BCG cultures (harboring the Mtb transcription factor overexpression library; OD_600_ ≈ 1.0, 1 × 10^10^ CFUs) were spread on ACA-containing 7H10plates and incubated at 37 °C for 4 weeks. Colonies were recovered and propagated in 7H9 broth containing 9 μg/mL ACA. Genomic DNA was isolated using the DNeasy Blood & Tissue Kit (Qiagen) according to the manufacturer's instructions. Whole-genome sequencing was performed using Illumina technology, with sequencing libraries prepared using the SureSelectXT Target Enrichment System Paired-End Sequencing Library (Agilent Technologies, Inc. Santa Clara, CA, USA) according to the manufacturer's instructions at Shanghai Biotechnology Corporation. Sequencing reads were aligned to the *M. bovis* BCG Pasteur 1173P2 reference genome using the Burrows–Wheeler aligner (v0.7.10) with default parameters (25). The insertion mutation (*BCG_0932*) identified by whole-genome sequencing was validated using Sanger sequencing.

### 2.4 Construction of *acasR*, *fadD9*-deletion mutant of *M. bovis* BCG, and *mbtB*-deletion mutant of *M. tuberculosis* H37Ra

Knockouts of *acasR* and *fadD9* in *M. bovis* BCG, and *mbtB* in *M. tuberculosis* H37Ra, were performed as previously described, with some modifications (32). A pMind-derived suicide plasmid carrying a hygromycin resistance gene was constructed, and the reporter gene *lacZ* was inserted as a selection marker. The primers used for gene knockout are listed in Supplementary Table 1. Genomic DNA from allelic exchange mutants in which *acasR*, *fadD9*, or *mbtB* were knocked out was identified by PCR analysis using primers for *acasR*, *fadD9-NTD*, *mtbB*, and the hygromycin gene (Supplementary Table 1).

### 2.5 ACA sensitivity assays

The recombinant strains were grown in 7H9 medium containing 30 μg/mL Kan at 37 °C for a week. Cells were cultured to an OD_600_ between 1.5 and 2.0, and each culture was diluted in 100 mL fresh 7H9 broth to an OD_600_ of approximately 0.15 with or without the indicated ACA concentration. Aliquots were extracted at the indicated time points and plated on 7H10 medium (supplemented with 10% OADC enrichment and 0.2% glycerol) to determine the number of colony-forming units (12).

### 2.6 Electrophoretic mobility shift assay (EMSA)

EMSAs were performed as previously described, with several modifications (33). The upstream regulatory sequences of *acasR* (350 bp), *fadD9* (350 bp), and *Rv1219* (274 bp) were amplified by PCR using specific primers (Supplementary Table 1). For EMSAs, DNA substrates were incubated at 25 °C for 30 min with various amounts of proteins in a total volume of 10 μL of EMSA buffer (50 mM Tris-HCl, pH 7.5; 10 mM MgCl_2_, 1 mM DTT, and 100 mM NaCl). The mixtures were subjected to 5% native polyacrylamide gel electrophoresis in 0.5 × Tris-borate-EDTA buffer. Electrophoresis was performed at 150 V and 25 °C until the bromophenol blue band reached the bottom of the gel. The gel was stained in EB for 2 min, and images were captured using Gel Doc^TM^ XR^+^ with Image Lab^TM^ software (Bio-RAD).

### 2.7 ChIP assays

Chromatin immunoprecipitation (ChIP) was performed as previously described, with modifications (32). In general, *acasR*-overexpression and *acasR*-knockout *M. bovis* BCG strains were grown in 100 mL 7H9 medium up to an OD_600_ of 1.0, fixed with 1% formaldehyde for 20 min, and terminated with 0.125 M glycine for 5 min. Cross-linked cells were harvested and resuspended in 1 mL Tris-buffered saline containing Tween-20 and Triton-X 100 (20 mM Tris-HCl [pH 7.5], 150 mM NaCl, 0.1% Tween 20, and 0.1% Triton X-100). The sample was sonicated on ice, and the average DNA fragment size was determined to be approximately 0.5 kb. A 100-*μ*L sample of the extract was saved as the input fraction, whereas the remaining 900 μL was incubated with 10 μL of antibodies against AcasR (Raised against *M. tuberculosis* H37Rv AcasR, this antibody fully recognizes the identical BCG homolog) or preimmune serum under rotation for 3 h at 4 °C. The complexes were immunoprecipitated with 20 μL 50% protein A agarose for 1 h under rotation at 4 °C. The immunocomplex was recovered by centrifugation and resuspended in 100 μL TE (20 mM Tris–HCl, pH 7.8; 10 mM EDTA; 0.5% SDS). Crosslinking was reversed through a water bath at 65 °C for 6 h. The DNA samples of the input and ChIP were puriﬁed, resuspended in 50 μL TE, and analyzed by PCR with Platinum Taq (Invitrogen). The application protocol included one denaturation step at 95 °C for 5 min, followed by 25 cycles of 1 min at 95 °C, 30 s at 60 °C, and 30 s at 72 °C. The PCR products were subjected to 1.5% agarose gel electrophoresis in 1 × Tris acetate-EDTA buffer at 100 V for 30 min.

### 2.8 *β*-Galactosidase activity assays

*β*-Galactosidase activity assays were performed as previously described, with several modifications (32). Experiments on *M. bovis* BCG were conducted by constructing an operon-lacZ fusion based on the expression vector pMV261. Target and control promoters were cloned into the pMV261 backbone. The reporter gene *lacZ* was then cloned behind the promoters. The plasmids were transformed into the *acasR*-knockout and wild-type *M. bovis* BCG strains to obtain recombinant reporter strains (Supplementary Table 2). All recombinant strains were grown in 7H9 medium at 37 °C until the log phase was reached. The bacterial cells were harvested and washed twice with phosphate-buffered saline (PBS). *β*-galactosidase measurements were performed as described previously (19).

### 2.9 Transcriptomic analysis

The *fadD9*-knockout, wild-type, and ACA-treated (*M. bovis* BCG wild-type strain grown in 7H9 medium to the exponential phase [OD_600_ ≈ 0.6] and treated with 15 μg/mL ACA for 26 h). *M. bovis* BCG strains grown to the exponential phase (OD_600_ ≈ 1.0) were harvested. Total RNA was extracted and purified using the RNeasy Mini Kit (Cat. #74106; Qiagen) according to the manufacturer's instructions. RNA integrity was determined using the RNA integrity number generated using an Agilent Bioanalyzer 2100 (Agilent Technologies). Total RNA was quantitated using the Qubit®3.0 Fluorometer and NanoDrop One spectrophotometer. Strand-specific libraries were prepared using the SureSelect Strand-Specific RNA Component Kit (Catalog No. G9691B; Agilent) according to the manufacturer's instructions. Briefly, ribosomal RNA was removed from the total RNA using VAHTS RNA Clean Beads (Catalog No. N412-02; Vazyme Biotech Co., Ltd., China). Following purification, the mRNA was fragmented into small pieces using divalent cations at 94 °C for 8 min. Cleaved RNA fragments were copied into first-strand cDNA using reverse transcriptase and random primers. This was followed by synthesis of second-strand cDNA using DNA polymerase I and RNase H. These cDNA fragments were then subjected to an end repair process, followed by the addition of a single ‘A’ base and ligation of the adapters. The products were purified and enriched using PCR to create a final cDNA library. Purified libraries were quantified using the Qubit®3.0 Fluorometer (Life Technologies, USA) and validated with an Agilent 2100 bioanalyzer (Agilent Technologies) to confirm the insert size and calculate the mole concentration. Clusters were generated using cBot with the library diluted to 10 pM and sequenced on an Illumina NovaSeq 6000 (Illumina, USA). Library construction and sequencing were performed at Shanghai Sinomics Corporation. Raw sequencing reads were trimmed for adapters and low-quality bases using Trimmomatic. Clean reads were aligned to the *M. bovis* BCG Pasteur 1173P2 reference genome using HISAT2. Gene-level read counts were quantified using featureCounts. Differential expression analysis was performed using DESeq2. Genes with |log2(FC)| > 1 and adjusted *P*-value (FDR) < 0.05 were considered significantly differentially expressed. The Benjamini-Hochberg procedure was used to control the false discovery rate (FDR). Raw counts were normalized using DESeq2's median-of-ratios method. Principal component analysis (PCA) was performed on the variance-stabilized transformed (VST) data to visualize sample clustering. To focus on the most biologically relevant changes, only analytes with relatively higher absolute fold-changes (both upregulated and downregulated) were selected for heatmap display. The complete, unfiltered dataset is provided in the supplementary tables. The selected log₂ fold-change values (treatment/WT) were directly used to generate the heatmaps using the online tool ImageGP. In the heatmaps, red indicates higher abundance than WT, blue indicates lower abundance than WT, and the color intensity reflects the magnitude of the log₂ fold-change.

### 2.10 Lipidomic analysis

The *fadD9*-knockout, wild-type, and ACA-treated (*M. bovis* BCG wild-type strain grown in 7H9 medium to exponential phase [OD_600_ ≈ 0.5] and treated with 10 μg/mL ACA for 2 days) *M. bovis* BCG strains grown in 7H9 medium to the exponential phase (OD_600_ ≈ 1.2) were harvested. Next, the cell pellets were washed twice with PBS. Cell pellets were subsequently resuspended in methanol/water (1:1, v/v; 0.8 mL) and homogenized using a small steel ball to extract cell lipids. The homogenate (0.8 mL) was transferred to a 2 mL sample bottle and mixed with 0.8 mL of chloroform, followed by vortexing for 30 s. The samples were sonicated in an ice-water bath for 10 min. After standing for 30 min, the different phases were separated by centrifugation at 2000rpm for 10 min. The underlayer phase (chloroform layer) was transferred to a new sample bottle and evaporated to dryness under vacuum. The upper phase was re-extracted by adding 1 mL chloroform/methanol (2/1, v/v), as described above. The upper phase was discarded, and both organic phases were combined and dried under vacuum. The lipid residues were dissolved in isopropanol/methanol (1:1, v/v; 0.3 mL; vortexed for 30 s and sonicated for 3 min) and centrifuged at 12000rpm for 15 min at 4 °C. The supernatants (200 μl) were then analyzed by LC-MS. The analysis was performed on a Q Exactive hybrid quadrupole-Orbitrap mass spectrometer. Chromatographic conditions were: column temperature 45 °C; mobile phase A consisting of acetonitrile/water (60:40, v/v) with 10 mmol/L ammonium formate and 0.1% formic acid; mobile phase B consisting of acetonitrile/isopropanol (10:90, v/v) with 10 mmol/L ammonium formate and 0.1% formic acid; flow rate 0.4 mL/min; injection volume 5 µL. For mass spectrometry, in positive ion mode, the heater temperature was 300 °C, sheath, auxiliary, and sweep gas flow rates were 45, 15, and 1 arb, respectively, spray voltage 3.0 kV, capillary temperature 350 °C, S-Lens RF level 50%, and scan range m/z 200–1800. In negative ion mode, parameters were identical except for a spray voltage of 2.5 kV. Structural analysis of lipids was performed at MajorBio (Shanghai, China). lipidomics analyses were performed as described previously (31). Briefly, raw data were processed using Progenius QI for peak alignment, generating a peak list that included retention time, mass-to-charge ratio (m/z), and peak area for each sample. Metabolite identification was performed against several databases, namely NIST, Lipidmaps, and the Mtb LipidDB, with a mass tolerance of 5 ppm. Six biological replicates were analyzed per sample. To identify differentially abundant metabolites between the two groups, univariate analysis was conducted using Student's t-test, calculating both fold change and statistical significance (*P*-value). Heatmaps were generated using ImageGP according to the methods described for transcriptome heatmap construction.

### 2.11 Quantification of iron concentrations

Iron concentrations were measured using a colorimetric ferrozine-based assay as previously described, with several modifications (29). Mycobacterial cultures were grown to an OD_600_ ≈ 0.5. The appropriate concentration of ACA (DMSO as a negative control) was then added. Next, cultures were incubated for up to 2 days to an OD_600_ of 1.0. Mycobacterial cultures were harvested by centrifugation, washed twice with ice-cold PBS, and resuspended in 1 mL of 50 mM NaOH. The suspension was first vortexed vigorously for 5 min, then sonicated for 12 min until the lysate became visually clear. This combined chemical-physical lysis effectively disrupts both cell walls and membranes. The lysate was centrifuged at 10,000 × *g* for 15 min at 4 °C to separate the soluble fraction (supernatant) from the insoluble pellet (containing cell debris, membrane fragments, and precipitated material). Both fractions were collected for iron quantification as described below. Because the lysis is complete, these fractions are defined operationally, and all comparisons between ACA-treated and control groups are based on relative changes under identical processing conditions. To quantify bound iron, the lysate (0.1 mL) was mixed with 10 mM HCl (0.1 mL) and 0.1 mL of iron-releasing reagent (1.4 M HCl + 4.5% (w/v) aqueous solution of KMnO_4_; 1/1) and incubated at 60 °C for 2 h. After cooling, 0.03 mL iron-detection reagent (6.5 mM ferrozine + 6.5 mM neocuproine + 2.5 M ammonium acetate + 1M ascorbic acid in water) was added. Next, absorbance was measured at 550 nm. The total free iron concentration was determined as described above, without the addition of an iron-releasing reagent. The free reduced iron concentration was measured as the total free iron concentration without the addition of ascorbic acid to the iron detection reagent. Iron concentrations were determined based on a standard curve obtained with increasing concentrations of ferric chloride and normalized to the protein content.

### 2.12 Statistical analysis and reproducibility

Each treatment was performed with three biological replicates or six biological replicates (Lipidomic analysis). Data are presented as mean ± standard deviation (SD). to ensure reproducibility. Data were analyzed using GraphPad Prism 8.0 software or Excel. Unpaired Student's t-tests were used to analyze differences between the treated and control groups.. Statistical signiﬁcance was deﬁned as *P ≤ 0.05, **P ≤ 0.01, and ***P ≤ 0.001.

## 3 Results

### 3.1 Whole-genome sequencing reveals that *acasR* mutation confers ACA resistance

Based on our previous finding that ACA kills *M. tuberculosis* by simultaneously targeting CpsA1 and CpsA2, we investigated the regulatory mechanisms underlying mycobacterial susceptibility to ACA. To this end, we constructed a transcription factor overexpression library. Transformants were plated on 7H10 agar plates containing 4 × and 8 × the MIC of ACA. A single resistant colony (BCG_R01) was obtained after one month of incubation. This colony showed a 3-fold higher MIC in 7H9 medium than that of the wild-type strain (9 μg/mL VS 3 μg/mL) (Table 1). Sequencing revealed that this strain harbors an overexpression vector for CarD (Rv3583c). The plasmid was extracted and subsequently retransformed into the wild-type BCG strains. However, the recombinant strains did not exhibit ACA resistance. Therefore, we hypothesized that the resistance observed in the original isolate resulted from genomic mutations rather than from CarD overexpression.

**Table 1:** The antibacterial activity of ACA against the selected microorganisms.

Strains	MIC (μg/mL)	MIC (μM)
*M. bovis* BCG Pasteur 1173P2	3	17.7
ACA-resistant *M. bovis* (BCG-R01)	9	53.1
*acasR* knockout *M. bovis* (Δ*acasR*)	6	35.4
*acasR* complemented *M. bovis*	2.5	14.75
*fadD9* knockout *M. bovis* (Δf*adD9*)	1.5	8.85
*fadD9* complemented *M. bovis*	4	23.6

**Figure 1:**
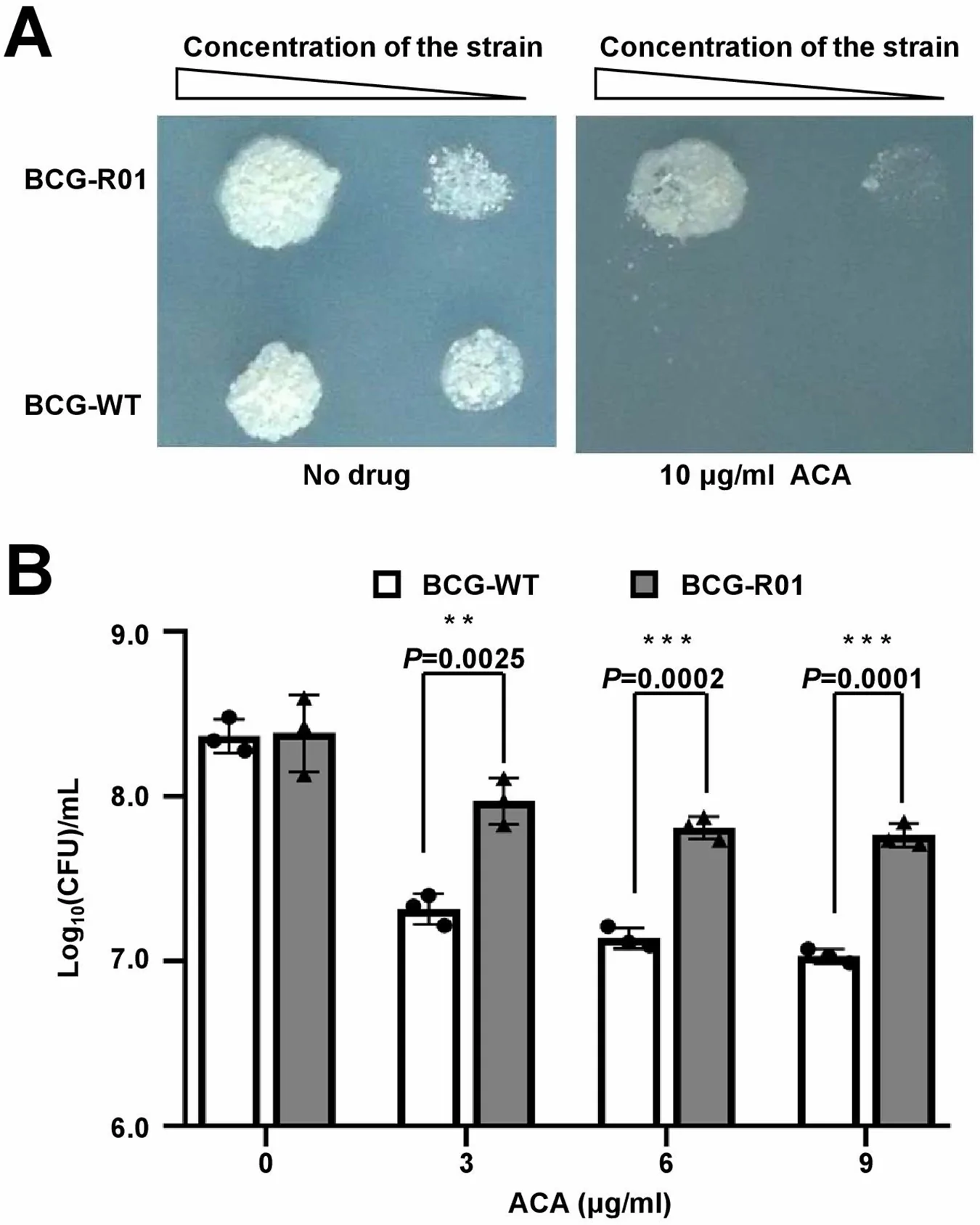
Assays for the resistance of *M. bovis* BCG mutant BCG-R01 to ACA. The wild-type and mutant strains were grown on solid agar 7H10 medium and in liquid 7H9 medium. **(A)** Strains were diluted 10 ×  to different concentrations, and equal amounts of culture were spotted on 7H10 plates with (right panel) or without 10 μg/mL ACA (left panel). **(B)** Assays for the resistance of BCG-R01 to ACA. The mycobacterial strains were grown in 7H9 medium containing 0 (control), 3, 6, and 9 μg/mL ACA at 37 °C for 7 days, and CFUs were measured. Error bars, SD (*n* = 3).

We further measured the growth of *M. bovis* BCG-R01 and wild-type BCG (BCG-WT) on the surface of 7H10 media with or without ACA. As shown in Figure 1A, no obvious difference in growth was observed among the BCG_R01 and wild-type strains in the absence of ACA (Figure 1A, left panel). In contrast, the BCG_R01 strain grew considerably better than the wild-type strain under 10 μg/mL ACA treatment (Figure 1A, right panel). In addition, we tested the resistance of BCG-R01 cells to ACA in the 7H9 medium containing various concentrations of ACA. As shown in Figure. 1B, BCG-R01 grew significantly faster than BCG-WT in 7H9 medium with 3 (*P* = 0.0025), 6 (*P* = 0.002), or 9 (*P* = 0.0001) μg/mL ACA. No obvious difference in the growth of the BCG-WT and BCG-R01 strains was observed in 7H9 medium in the absence of drugs (Figure 1B). These results strongly suggest that BCG-R01 is more resistant to ACA than the BCG-WT.

To identify mutations associated with resistance, whole-genome sequencing was performed on both BCG_R01 and the parental *M. bovis* BCG strain using Illumina sequencing technology (Supplementary Table 3). The BCG_R01 genome contained a C insertion at position 37 within the *BCG_0932* (*acasR*) gene, which encodes a MarR family transcriptional regulatory protein (Table 2). This insertion, which was further validated by Sanger sequencing, resulted in a frameshift mutation that terminated earlier at position 46. In addition to *acasR*, the mutant strain BCG_R01 contained other mutation sites, such as a frameshift mutation of PE_PGRS28 (BCG_1513c) and a single-nucleotide mutation in glycerol kinase (*GlpK*) (Table 2). Overexpression of *pe_pgrs28* or *glpK* in AR01 revealed that neither PE_PGRS28 nor GlpK restored the sensitivity of AR01 cells to ACA.

**Table 2:** Single-nucleotide polymorphisms or insertion/deletion mutations in the *Mycobacterium bovis* BCG pasteur 1173P2 R01(ACA-resistant) isolate.

Sample name	InDel/SNP description	Locus tag	Definition	SNP/Indel class	Codon_Change	AA change	Gene length
BCG_R01	G1009426GC	BCG_0932/Rv0880	MarR family transcriptional regulator	FRAME_SHIFT	-/C	−13?	432bp
BCG_R01	G1662465GC	BCG_1513c/ Rv1452c	PE-PGRS family protein PE_PGRS28	FRAME_SHIFT	-/G	−261?	2226bp
BCG_R01	C4112669T	BCG_3755c/Rv3696c	Probable glycerol kinase glpK	MISSENSE	gCg/gTg	A68V	1557bp

**Figure 2:**
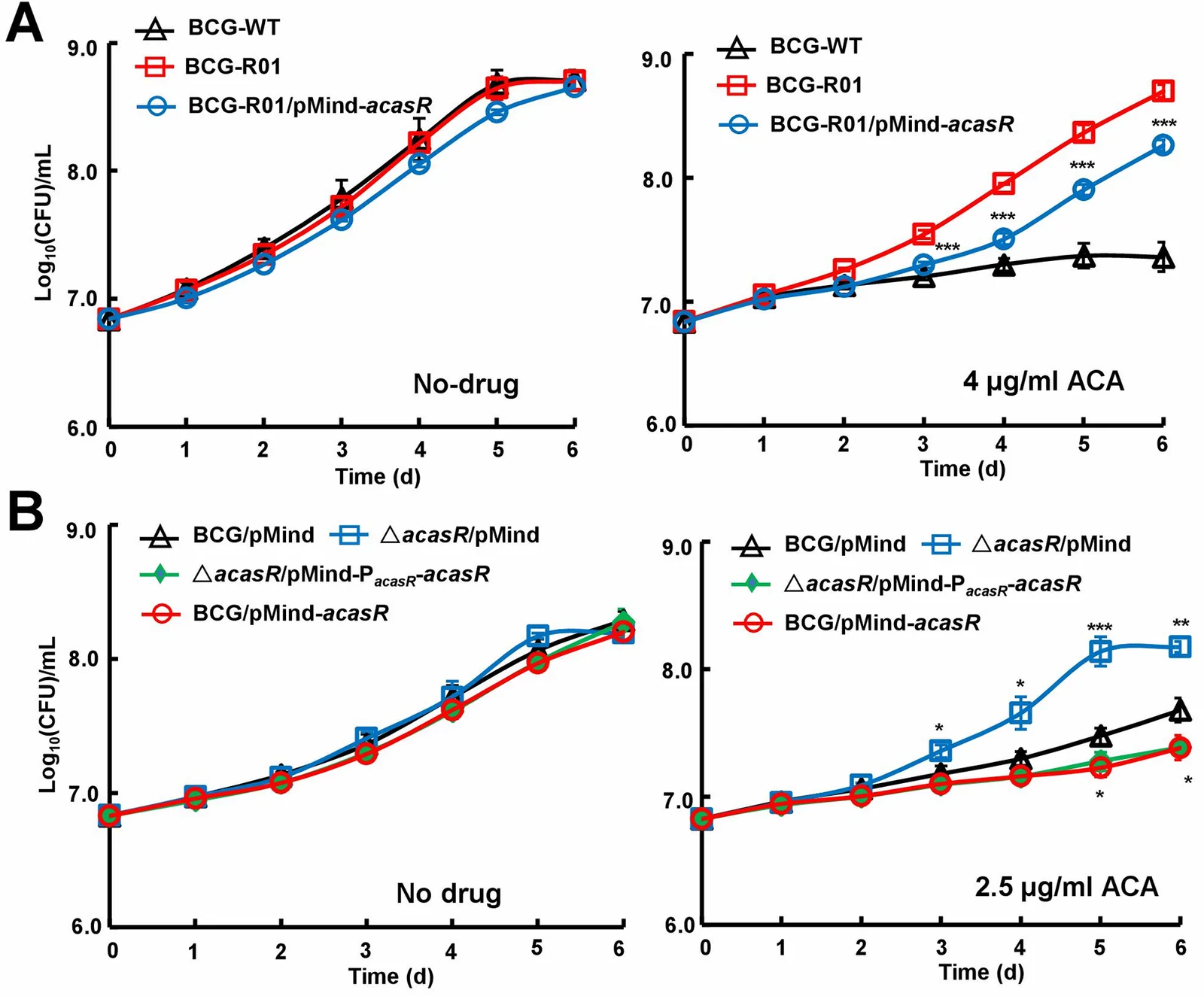
AcasR is responsible for the mycobacterial sensitivity to ACA. **(A)** The complemented strain BCG-R01/pMind-*acasR* regained sensitivity to ACA. Mycobacterial strains were grown in 7H9 medium with or without 4 μg/mL ACA at 37 °C for 6 days. Samples were extracted at the indicated time points, and CFU counts were performed. **(B)** Wild-type, *acasR*-knockout, complementary, and *acasR*-overexpressing mycobacterial strains were grown in 7H9 medium with or without 2.5 μg/mL ACA at 37 °C for 6 days. ACA concentrations were normalized to the MIC of each strain*.*Samples were extracted at indicated time points, and CFU counts were performed. Error bars, SD (*n* = 3).

Further complementation experiments confirmed that the mutation of *acasR* is responsible for mycobacterial resistance to ACA. When the mutant strain BCG_R01 was transformed with a pMind vector overexpressing wild-type *acasR*, the complemented strain showed partial sensitivity to ACA (Figure 2A). To further verify that the mutation of *acasR* is responsible for mycobacterial resistance to ACA, we constructed an *acasR*-knockout (Δ*acasR*/pMind) *M. bovis* BCG strain (Supplementary Figure 1A), its complemented strain (Δ*acasR*/pMind-P*_acasR_*-*acasR*), and an *acasR*-overexpressing strain (BCG/pMind-*acasR*). As shown in Figure 2B, the *acasR*-knockout BCG strain grew significantly better than the wild-type BCG strain in 7H9 medium containing 2.5 μg/mL of ACA (*P* < 0.001 on day 5). This phenotype was reversed in the complemented strain (Figure 2B, right panel). Under the same conditions, the *acasR*-overexpressing strain was significantly inhibited compared to the wild type (Figure 2B, right panel; *P* = 0.0109 on day 5). Compared with the wild-type strain, the *acasR* knockout strain exhibited a two-fold higher MIC of ACA (6 μg/mL vs. 3 μg/mL). In contrast, the complementation strain showed a slightly lower MIC of 2.5 μg/mL, which may be attributed to the use of an overexpression plasmid for complementation (Table 1). No obvious differences were observed in the growth of wild-type, *acasR*-knockout, *acasR*-complemented, and *acasR*-overexpressing *M. bovis* BCG strains in the 7H9 medium without drugs (Figure 2B, left panel). The same results were obtained when these strains grew on the surface of 7H10 medium with or without 12 μg/mL ACA (Supplementary Figure 2A). These results suggest that the *acasR* mutation leads to mycobacterial resistance to ACA.

### 3.2 AcasR binds the promoter of fatty acid CoA ligase (FadD9) encoding gene *Rv2590*

Orthologs of BCG_0932 (AcasR) were identified based on the sequence similarity and conservation of adjacent genes. The BCG_0932 region was highly conserved among *M. bovis* BCG, *M. tuberculosis* H37Rv, and *M. tuberculosis* H37Ra (100% amino acid identity over the entire length of the protein) (Supplementary Figure 3). In *M. tuberculosis* H37Rv, AcasR is encoded by *Rv0880*, which was previously reported as a MarR family protein (8).

**Figure 3:**
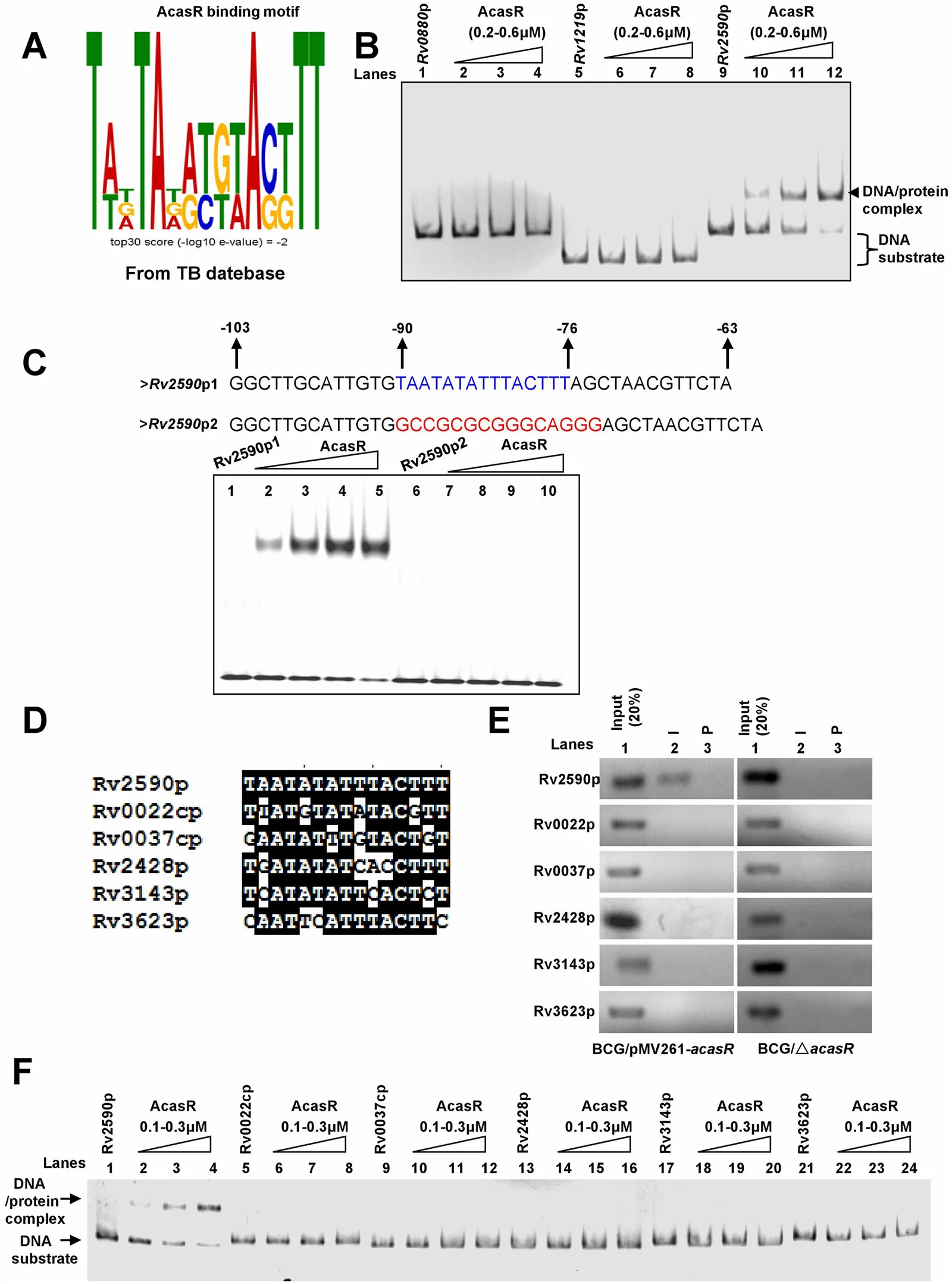
AcasR binds the promoter of fadD9. **(A)** The predicated AcasR binding motif (from the TB database). **(B)** EMSAs for the DNA-binding activity of AcasR on the upstream regions of *acasR* (*Rv0880*p), *Rv1219* (Rv1219p), and *fadD9* (*Rv2590*p). **(C)** EMSAs for DNA substrates with (*Rv2590*p1) or without (*Rv2590*p2) the AcasR-binding motif sequence. Each DNA substrate was co-incubated with 0.2–0.6 μ M AcasR. **(D)** The *acasR*-binding motif (TAATATATTTACTTT) was used to search the intergenic regions between *M. tuberculosis* H37Rv open Reading frames. A custom in-house Perl/Python script was used to scan the *M. tuberculosis* H37Rv genome. For each annotated ORF, the 500 bp upstream region (relative to the start codon) was extracted. The scan allowed 0 to 4 nucleotide mismatches, without gaps or insertions. The conserved sequence is highlighted by black backgrounds. **(E)** ChIP assays for the association of AcasR with the predicated target genes. ChIP using preimmune (P) or immune serum (I) against AcasR. The DNA samples for the input and ChIP were puriﬁed and resuspended in 50 μl TE. DNA recovered from the immunoprecipitates was ampliﬁed with primers speciﬁc for the predicated target genes. **(F)** EMSAs for the DNA-binding activity of AcasR on the predicated DNA substrates. *Rv2590*p was used as a positive control. Either DNA substrate was co-incubated with 0.1–0.3 μM AcasR protein.

According to TBDB (tbdb.org (7),), *Rv1219c* and *Rv2590* (fatty acid CoA ligase-encoding gene *fadD9*) may be the target genes of AcasR (20, 24) and the binding motif (Figure 3A). Therefore, EMSA was conducted to evaluate the binding activity of AcasR to *Rv1219c*p (Rv1219c upstream 200 bp + open reading frame 74 bp) and *Rv2590*p (Rv2590 upstream 350 bp) *in vitro*. Clearly shifted bands were observed when the Rv2590 promoter DNA substrates were co-incubated with increasing amounts of AcasR (Figure 3B, lanes 9–12). However, AcasR could not bind to the Rv1219c promoter DNA or its own promoter, Rv0880p (Figure 3B, lanes 1–8). Thus, we analyzed the Rv2590 promoter DNA sequence to identify other target genes of AcasR. A motif (5′-TAATATATTTACTTT-3′) was detected in AcasR-binding motif (Figure 3A), from position −90 to −76 in the coding strand (Figure 3C). Furthermore, EMSA confirmed the significance of the motif for specific recognition by AcasR. DNA substrate mutants were synthesized (Figure 3C, upper panel) and EMSAs were conducted (Figure 3C, lower panel). Compared to inherent *Rv2590*p1 (Figure 3C, lanes 1–5), AcasR lost its ability to bind to *Rv2590*p2 (Figure 3C, lanes 6–10), in which the specific binding motif was replaced by random sequences. These results suggest that this binding motif is essential for AcasR binding. The intergenic regions of the *M. tuberculosis* genome were searched based on the sequence motif, and five potential target genes were identified (Figure 3D).

ChIP assays confirmed the binding of AcasR to *Rv2590*p *in vivo*. As shown in Figure 3E, AcasR was cross-linked to *Rv2590*p. *Rv2590* promoter DNA was particularly detected by immunoprecipitation using the specific AcasR antiserum in the AcasR-overexpressing BCG strain (Figure 3E, left panel, lane 2), whereas it could not be co-precipitated under the same conditions in the *acasR*-knockout BCG strain (Figure 3E, right panel, lane 2). In contrast, the preimmune serum failed to precipitate any detectable DNA (Figure 3E, left panel, lane 3). *Rv0880*p, the promoter of *acasR* used as a negative control, was not captured by the AcasR antiserum. This indicates that AcasR specifically binds to *Rv2590*p *in vitro* and *in vivo*. Surprisingly, both EMSA and ChIP assays confirmed that AcasR could not bind to all potential target genes *in vivo* and *in vitro* (Figures 3E,F).

The above ﬁndings strongly suggest that AcasR can bind with the promoter of Rv2590, which encodes the fatty acid CoA ligase FadD9.

### 3.3 AcasR negatively regulates *fadD9* expression

**Figure 4:**
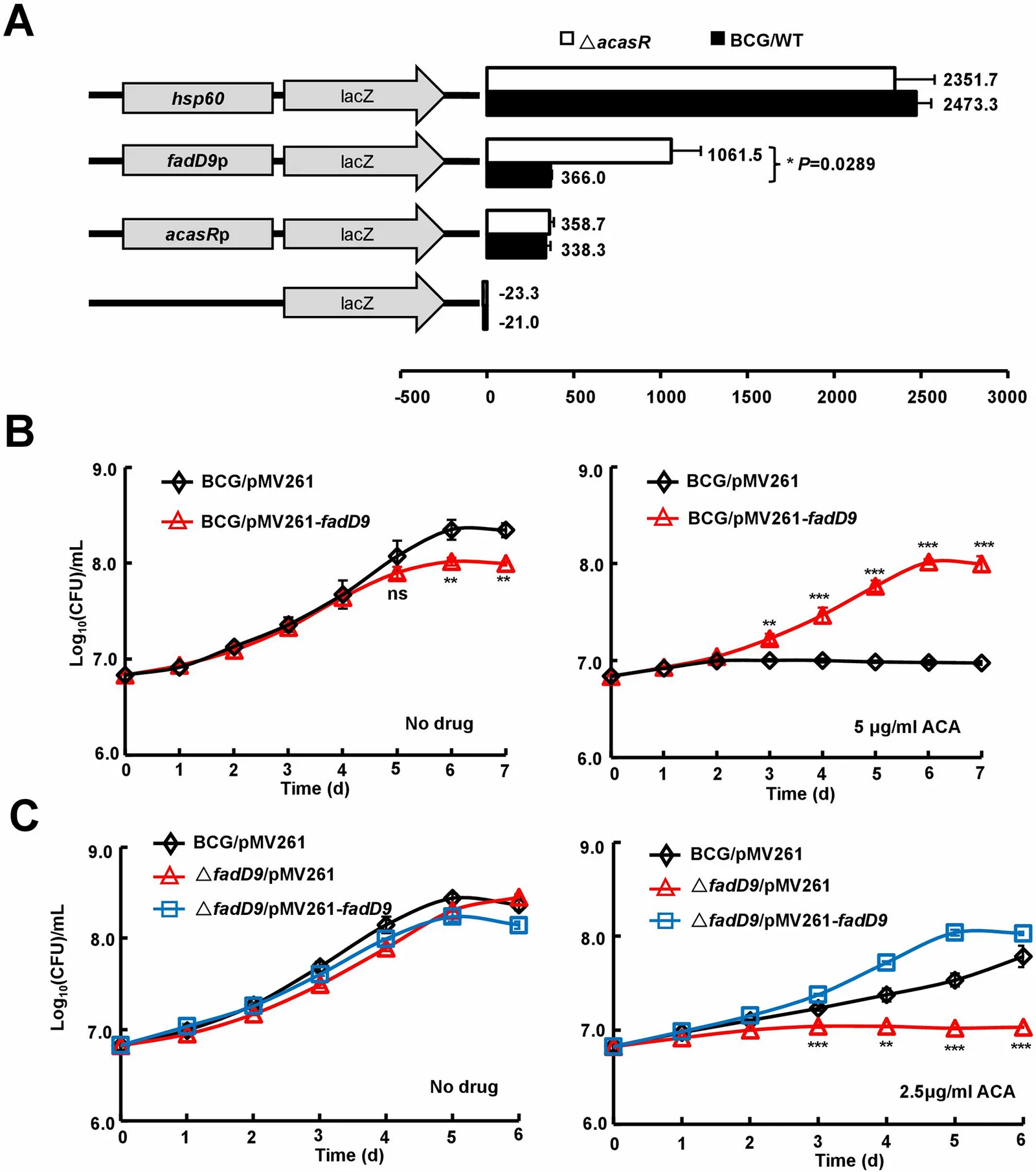
Fadd9 is negatively regulated by acasR and is responsible for the resistance of mycobacteria to ACA. **(A)** *β*-galactosidase activity assays. The effect of AcasR on gene expression was evaluated by constructing the *fadD9*p-lacZ plasmid. The activity of *β*-galactosidase was further examined in the wild-type *M. bovis* (BCG/WT) and *acasR* knockout (ΔacasR) strains. The data are presented as Miller units (right panel). Left column: schematic representation of each clone used to generate recombinant strains. Two-tailed Student's t-tests were used for statistical analysis; *P* ≤ 0.05 was considered statistically significant. Null promoter-lacZ, *hsp60*-lacZ, and *acasR*p-lacZ were used as controls. Data are presented as the averages of three independent biological experiments. The *P*-values of the results (*P* = 0.0289) are indicated by single asterisks (*) on top of the column in the figure. **(B)** Wild-type, *fadD9*-overexpressing, **(C)** *fadD9*-knockout, and complementary mycobacterial strains were grown in 7H9 medium with or without ACA. The concentration of ACA is shown in the figure. Samples were extracted at indicated time points, and CFU counts were performed. Error bars represent SD (*n* = 3). The *P*-values were calculated by unpaired two-tailed Student's t-test using GraphPad Prism 8.

We constructed a series of *lacZ* or promoter-*lacZ* co-expression plasmids to examine the regulatory effect of AcasR on gene expression using *β*-galactosidase activity assays. As shown in Figure 4A, the strong promoter *hsp60* markedly promoted the expression of *lacZ* in both wild-type and *acasR*-knockout *M. bovis* BCG strains compared to the non-promoter *lacZ* plasmid, indicating that the reporter system was efficient. Strikingly, when *fadD9*p was used as the promoter of *lacZ*, the expression of *lacZ* was significantly upregulated in the *acasR*-knockout mutant *M. bovis* BCG strain compared to that in the wild-type strain (*P* = 0.0289). In contrast, no significant difference in the expression of *lacZ* was observed between the wild-type and *acasR*-knockout BCG strains when the negative control *acasR*p was used as a promoter. Taken together, these results indicated that AcasR is an inhibitor that negatively regulates the expression of *fadD9*.

### 3.4 FadD9 is responsible for the resistance of mycobacteria to ACA

To further elucidate the role of FadD9 in the resistance of mycobacteria to ACA, we constructed *fadD9*-overexpressing and *fadD9*-knockout *M. bovis* BCG strains (Supplementary Figure 1A) and a complemented strain (Δ*fadD9*/pMV261-*fadD9*). We further compared the differential ACA-resistance or ACA-sensitivity of these strains. In the absence of drugs, the *fadD9*-overexpressing BCG strain grew similarly to the wild-type strain in 7H9 medium during early logarithmic phase; however, a significant growth defect became evident from day 4 onward as the culture approached stationary phase (Figure 4B, left panel; *P* = 0.0064 on day 6). However, this strain grew significantly better than the wild type BCG strain following the addition of 5 μg/mL ACA (Figure 4B, right panel; *P* = 0.0005 on day 4). Similar results were observed when the strains grew on the surface of 7H10 medium with or without 12 μg/mL ACA. Moreover, the *fadD9*-overexpressing BCG strain grew even better than the *acasR*-knockout BCG strain in the presence of 12 μg/mL ACA (Supplementary Figure 2B). The growth of the *fadD9*-knockout strain, its complemented strain, and the wild-type BCG strain were similar in the absence of ACA (Figure 4C, left panel). With 2.5 μg/mL of ACA, the growth of the *fadD9*-knockout strain was more significantly inhibited than that of the wild-type strain (Figure 4C, right panel; *P* = 0.0002 on day 5). This inhibition phenotype was effectively rescued when *fadD9* was complemented by the *fadD9*-knockout strain (Figure 4C). Compared with the wild-type strain, the *fadD9* knockout strain exhibited a two-fold lower MIC of ACA (1.5 μg/mL vs. 3 μg/mL). In contrast, the complementation strain showed a slightly higher MIC of 4 μg/mL, which may be attributed to the use of an overexpression plasmid for complementation (Table 1). These results suggest that FadD9 is responsible for mycobacterial resistance to ACA.

### 3.5 ACA stress leads to intracellular iron deficiency in *Mycobacterium*

To further elucidate the mechanisms through which ACA kills mycobacteria, we conducted transcriptomic assays to compare the differential expression of genes between ACA-treated (15 μg/mL for 26 h) and untreated *M. bovis* BCG strains. The expression of several genes was upregulated or downregulated in the ACA-treated BCG strain (Supplementary Figure 4, Supplementary Table 4). It is worth mentioning that ACA treatment induces the expression of *fadD9* as shown in Supplementary Table 4 and as can be expected by the results reported in this manuscript.

**Figure 5:**
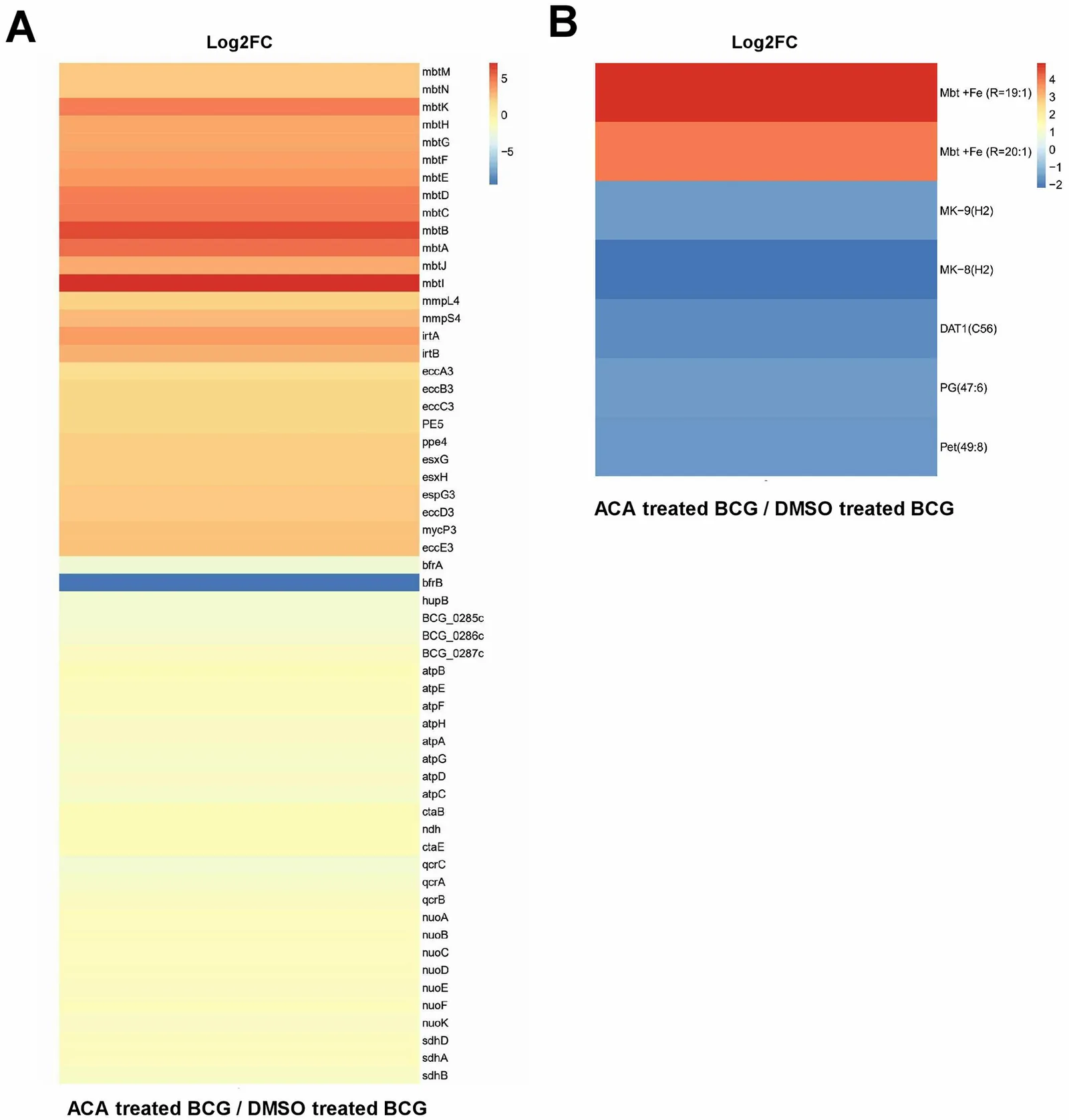
Transcriptomic and lipidomic assays for the effect of ACA on gene expression in *Mycobacterium bovis* BCG. **(A)** A heatmap of the ACA-triggered differential expression profile for siderophore synthesis-, transport-, iron storage-, and oxidative phosphorylation-related genes. **(B)** Heatmap representation of the most differentially present lipids in the wild-type and ACA-treated *M. bovis* BCG strains. Data are presented as log₂ fold-change (ACA treated BCG/DMSO treated BCG). Genes with significantly upregulated expression are shown in red. Those with significantly downregulated expression are shown in blue. All genes presented in the figure exhibited statistically significant differences between the treatment group and the control group (*P* < 0.05). The heatmap was generated using ImageGP as described in the Methods section.

Iron acquisition-associated genes were among those with the most highly upregulated expression. These included the mycobactin synthesis gene cluster (BCG_1407-1409c; BCG_2391c-2400c), siderophore secretion machinery Esx-3 (BCG_0322-BCG_0332), siderophore exporters (*mmpL4* and *mmpS4*), and iron importers (*irtA* and *irtB*). The expression of iron storage proteins (bfrA and bfrB) was substantially downregulated in the ACA-treated strain. In addition, the genes with significantly downregulated expression in the ACA-treated strains were primarily enriched in energy metabolism processes (ATP synthesis, ribosomal proteins, NADH dehydrogenation, and cellular respiration) (Figure 5A, Supplementary Table 4). These changes in gene expression were nearly identical to those previously reported under iron-deficient conditions (1, 23), suggesting that ACA stress induces intracellular iron deficiency in mycobacteria. Further, we conducted lipidomic analysis between ACA-treated (10 μg/mL for 2 days) and untreated *M. bovis* BCG strains. As shown in Figure 5B and Supplementary Table 5, mycobactin (Mbt + Fe) increased significantly (Mbt + Fe (*R* = 19:1), 30-fold; Mbt + Fe (*R* = 20:1), 15.9-fold) in the ACA-treated *M. bovis* BCG strain. Furthermore, the expression of the menaquinones MK-8 and MK-9 was downregulated approximately 4.5-fold and 2.8-fold, respectively. This was consistent with the transcriptomic data. Our multi-omics data collectively indicate that ACA treatment induces intracellular iron starvation and decelerates energy metabolism in mycobacteria.

**Figure 6:**
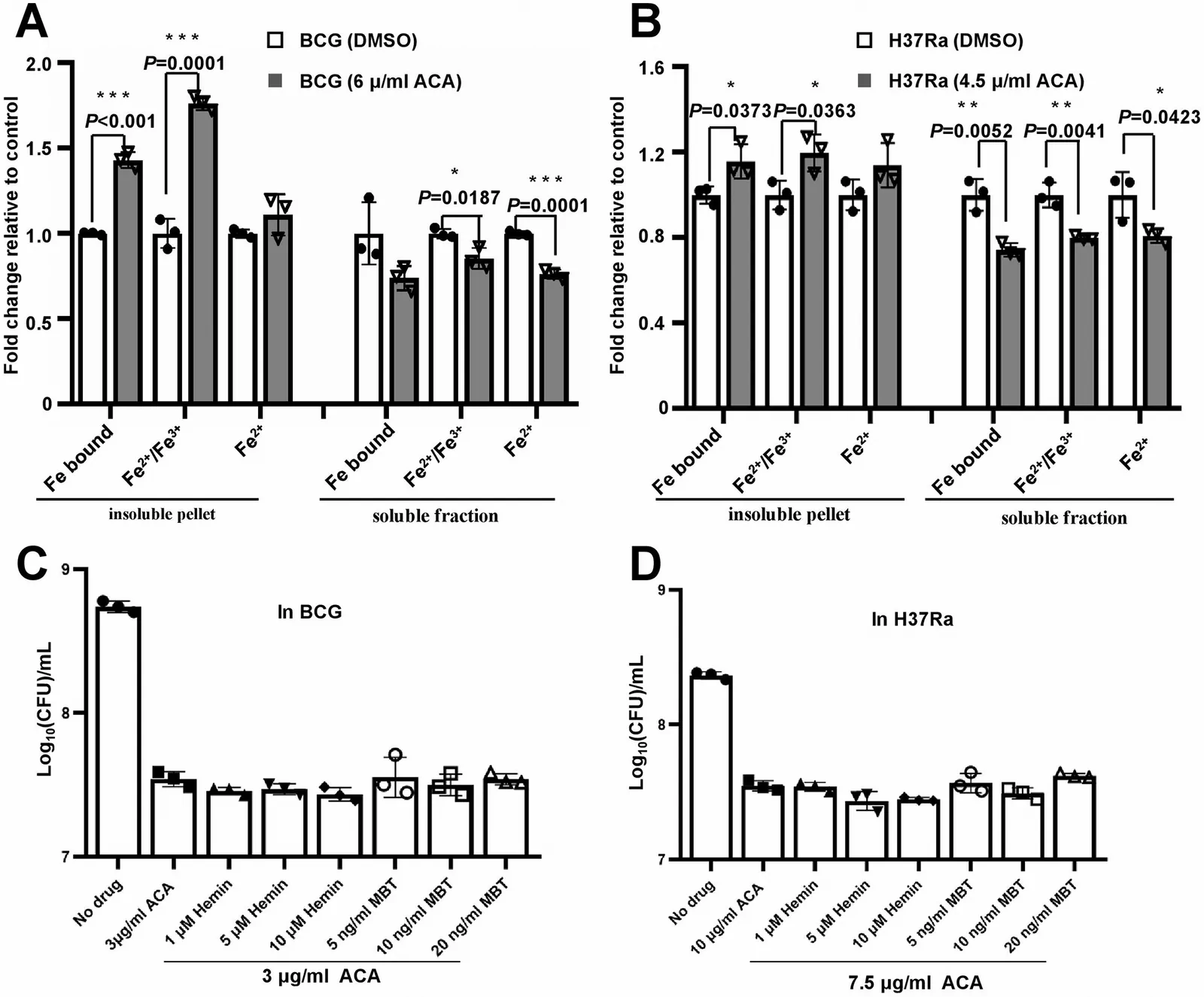
The effects of ACA on intracellular iron concentration or supplemental hemin (or mycobactin) on the ACA resistance in *Mycobacterium*. **(A)** Wild-type *M. bovis* BCG or *M. tuberculosis* H37Ra **(B)** were grown in 7H9 medium and treated with 6 μg/mL ACA or 4.5 μg/mL ACA for 2 days. Concentrations of protein-bound, total free (ferric + ferrous), and free ferrous iron were measured as described in the Methods. Iron concentrations are presented relative to untreated controls. **(C)** Wild-type *M. bovis* BCG or *M. tuberculosis* H37Ra **(D)** were grown in 7H9 medium and treated without or with ACA (or ACA + Hemin or ACA + mycobactin). The concentration of ACA is shown in the figure. Samples were extracted at indicated time points, and CFU counts were performed. Error bars, SD (*n* = 3).

To detect changes in iron concentrations in the cell envelope and cytoplasm of ACA-treated bacterial strains, the concentrations of bound, reduced, and oxidized free iron were measured in *Mycobacterium* cultures with or without ACA. In ACA-treated BCG strains, a significant increase in protein-bound and total free (ferric + ferrous) iron was observed on the insoluble pellet, whereas the total free iron and free ferrous (Fe^2^⁺) in the soluble fraction showed a marked decrease (Figure 6A). The changes in iron concentrations in the ACA-treated H37Ra strains, both in the insoluble pellet and soluble fraction, were nearly identical to those observed in the BCG strains (Figure 6B). Moreover, the inhibitory effect of ACA persisted despite supplementation with different concentrations of mycobactin or heme, demonstrating that ACA-mediated growth inhibition occurred through impaired iron (mycobactin) utilization rather than extracellular iron scarcity (Figures 6C,D).

*M. tuberculosis* H37Ra can utilize both mycobactin and heme as iron sources. Therefore, we sought to determine whether mycobacteria can develop resistance to ACA by bypassing the inhibition of the mycobactin utilization pathway if the mycobactin pathway is disrupted and H37Ra cells are supplemented with heme. To this end, we constructed the H37Ra/Δ*mbtB* strain (Supplementary Figure 1B). This strain exhibited significantly slower growth than wild-type mycobacteria under normal conditions (Supplementary Figure 5A and Figure 5B). However, growth of the *mbtB*-deficient strain was markedly restored when the medium was supplemented with 40 ng/mL mycobactin (Supplementary Figure 5A, second panel). Moreover, the growth of the H37Ra/Δ*mbtB* strain was partially recovered in the presence of 5 μM heme, although it remained slower than that of the wild-type strain (Supplementary Figure 5C). Under these conditions, we further added 12 μg/mL ACA. The *mbtB*-deficient strain exhibited better growth than the wild-type strain (Supplementary Figure 5D). This suggests that bacteria activate the heme utilization pathway upon losing the mycobactin-dependent iron acquisition pathway. As ACA primarily restricts mycobactin uptake by mycobacteria, the *mbtB*-deficient strain developed resistance to ACA in the presence of heme.

Taken together, these results suggest that ACA inhibits the transmembrane transport of iron-loaded carboxymycobactin, leading to iron accumulation on the membrane surface and decreased intracellular iron concentrations.

### 3.6 The deletion of *fadD9* reduces Mbt-Fe expression

**Figure 7:**
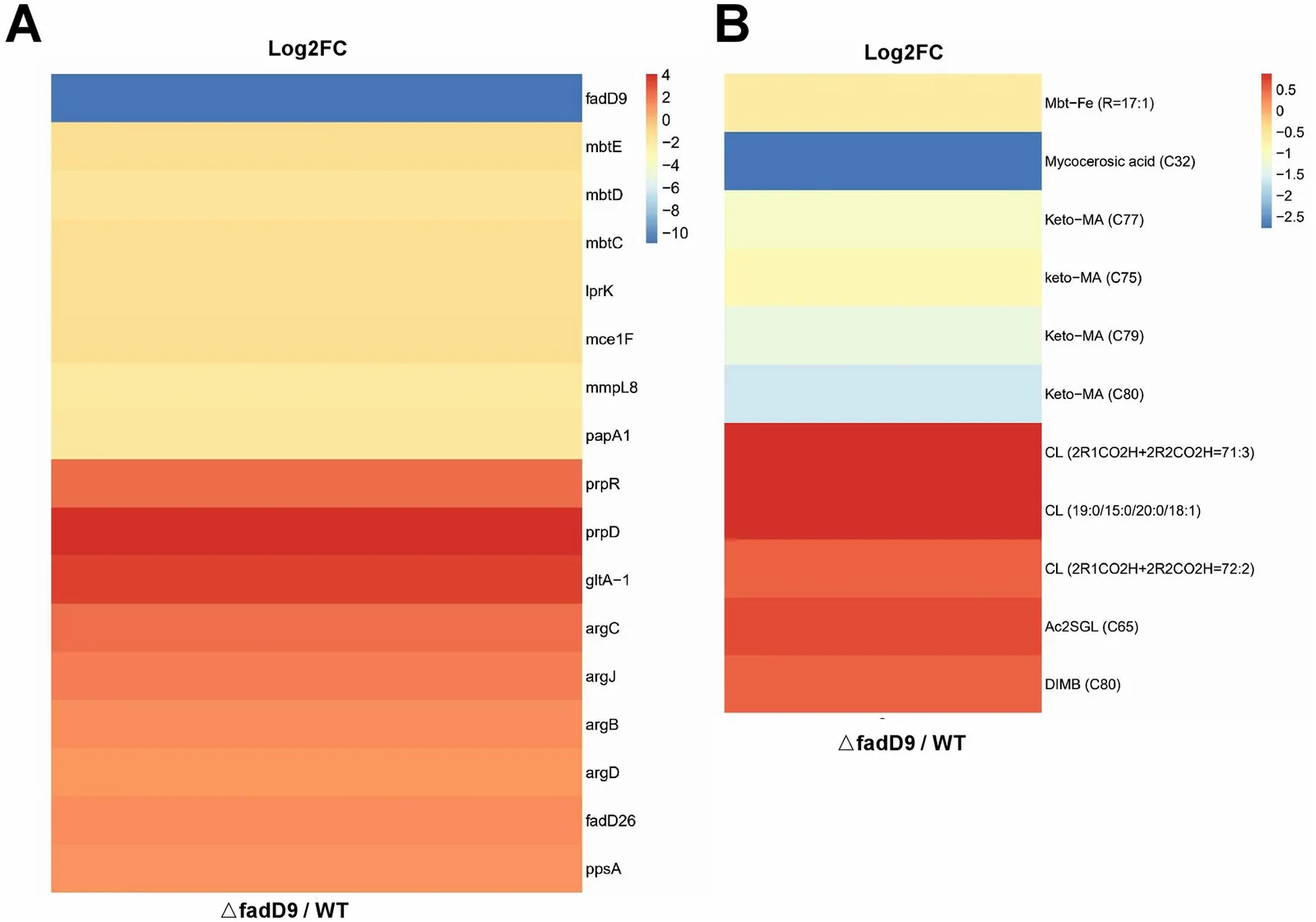
Transcriptomic and lipidomic assays for the effect of *fadD9* deletion on gene expression in *M. bovis* BCG. **(A)** Heatmap representation of the most differentially expressed genes in wild-type and *fadD9*-knockout *M. bovis* BCG strains. Red and green indicate genes with significantly upregulated and downregulated expression, respectively. **(B)** Heatmap representation of the most differentially expressed lipids in the wild-type and *fadD9*-knockout *M. bovis* BCG strains. Data are presented as log₂ fold-change (Δ*fadD9*/WT). Genes with significantly upregulated expression are shown in red. Those with significantly downregulated expression are shown in blue. All genes presented in the figure exhibited statistically significant differences between the treatment group and the control group (*P* < 0.05). The heatmap was generated using ImageGP as described in the Methods section.

To clarify why *fadD9*-knockout *M. bovis* BCG strain was more susceptible to ACA, we also conducted transcriptomic assays to compare differentially expressed genes between *fadD9*-knockout and wild-type *M. bovis* BCG strains. The expression of approximately 89 genes was upregulated or downregulated in the *fadD9*-knockout *M. bovis* BCG strain (Supplementary Figure 4B, Supplementary Table 6). Consistent with our expectations, *fadD9* had the most downregulated expression (0.0005-fold) in the *fadD9*-knockout *M. bovis* BCG strain (Figure 7A), suggesting that the transcriptomic data were credible. Furthermore, the mycobactin synthesis gene cluster (*mbtE*, 0.45-fold; *mbtD*, 0.34-fold; *mbtC*, 0.43-fold) was also downregulated in the *fadD9*-knockout *M. bovis* BCG strain (Figure 7A), suggesting that mycobactin synthesis was decreased. To validate this hypothesis, we conducted lipidomic analysis of *fadD9*-knockout and wild-type *M. bovis* BCG strains. As shown in Figure 7B and Supplementary Table 7, mycobactin synthesis (Mbt-Fe (R = 17:1) decreased significantly (0.64-fold), which was consistent with the transcriptomic data.

**Figure 8:**
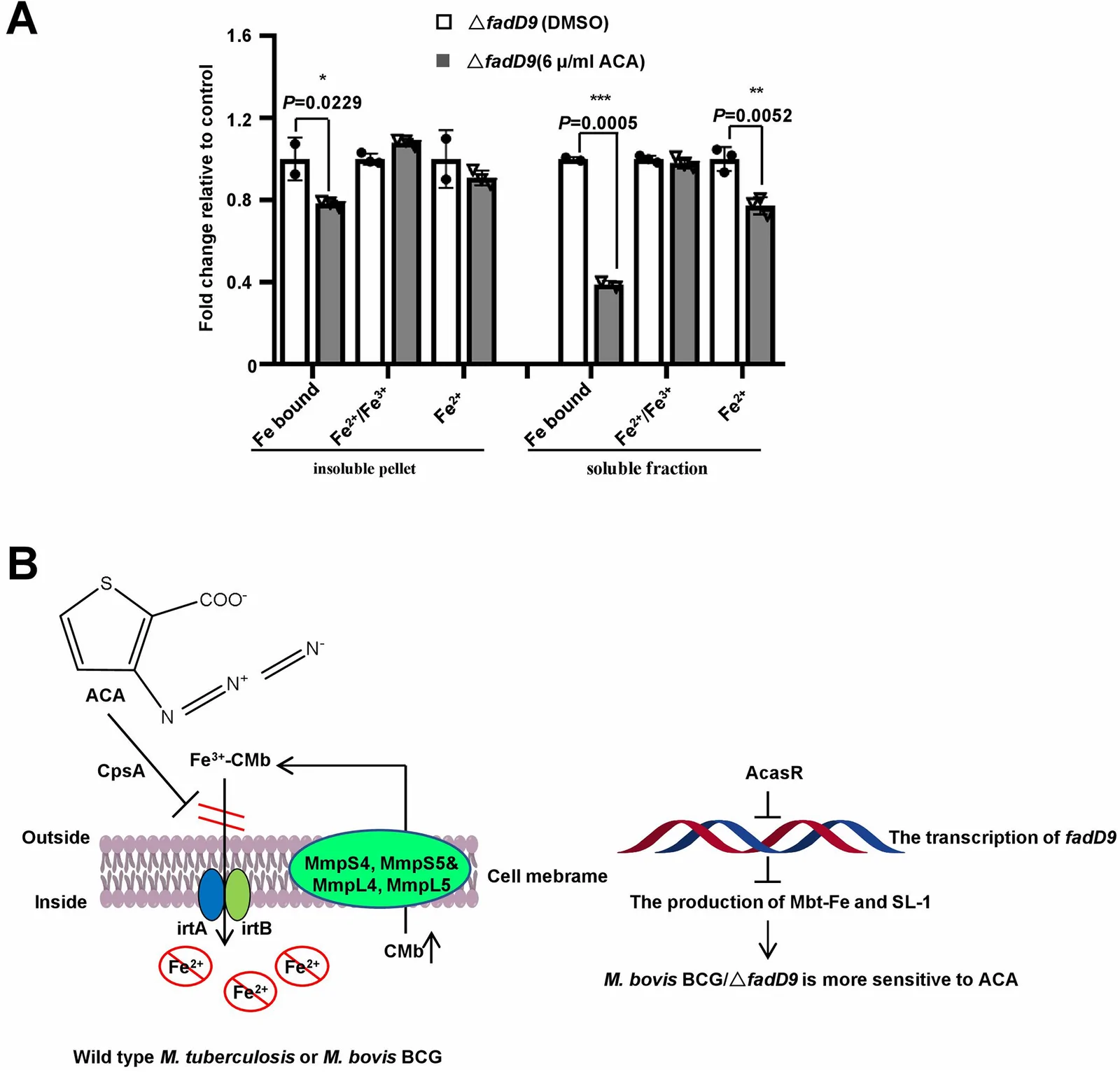
The mechanism through which acasR regulates sensitivity to ACA in *Mycobacterium* **(A)** wild-type and *fadD9*-knockout *M. bovis* BCG were grown in 7H9 medium and treated with 6 μg/mL ACA for 2 days. Protein-bound, total free (ferric + ferrous), and free ferrous iron concentrations were measured as described in the Methods. Iron concentrations are presented relative to untreated controls (DMSO). Error bars, SD (*n* = 3). **(B)** Schematic representation of the mechanisms through which AcasR regulates the sensitivity of *M. tuberculosis* to ACA. The schematic diagram of the cell membrane and nucleic acid structure is derived from the ChemDraw library.

Iron quantification assays further demonstrated that in the *fadD9*-deficient strain ACA treatment significantly decreased protein bound iron in the insoluble pellet fraction, and significantly reduced both protein-bound iron and free ferrous iron (Fe^2+^) in the soluble fraction. The protein-bound iron decreased by more than 2-fold (to less than 50% of the control level) in the soluble fraction (Figure 8A). These results suggest that the *fadD9*-knockout *M. bovis* BCG strain is more sensitive to ACA, which may be due to reduced mycobactin synthesis.

## 4 Discussion

ACA kills *M. tuberculosis* by targeting the CpsA proteins and impairing cell wall synthesis (13). However, the intrinsic regulatory mechanisms underlying mycobacterial sensitivity to ACA remain unclear. Through the screening of drug-resistant mutants, we found that ACA resistance is conferred by a frameshift mutation in the transcriptional regulator *acasR*.

In our previous study using *M. tuberculosis* H37Ra, ACA resistance was conferred by mutations in *cpsA2*, with a resistance frequency of approximately 10⁻^8^ and a 5-fold increase in MIC (13). In contrast, the current study using *M. bovis* BCG identified an *acasR* frameshift mutation conferring a 3-fold MIC increase at a much lower frequency (∼10⁻^10^). The discrepancy in resistance mechanisms and frequencies between the two studies likely reflects differences in the genetic backgrounds of the two mycobacterial species. While the precise molecular basis for these differences remains to be elucidated, our findings highlight that strain background can profoundly influence both the spectrum and the likelihood of acquired resistance mutations under selective pressure.

In this study, we observed that the Δ*acasR* knockout strain (MIC = 6 μg/mL, ∼2-fold increase) is less resistant to ACA than the R01 mutant (MIC = 9 μg/mL, ∼3-fold increase), despite both strains being in the same *M. bovis* BCG background and both lacking functional AcasR. This difference likely reflects their distinct origins: the Δ*acasR* strain is a clean laboratory knockout, whereas the R01 mutant was isolated under stepwise ACA selection, which may have enriched for additional adaptive mutations or physiological adaptations that enhance resistance beyond the direct effect of acasR disruption. Notably, because of their different MIC values, we used concentrations normalized to the MIC of each strain (0.4–0.45 × MIC) in the kill curve experiments (Figure 2), ensuring a fair comparison of bactericidal kinetics under equivalent relative selective pressures.

AcasR is a transcriptional regulator of the MarR gene family. The overexpression of *acasR* increases mycobacterial ACA susceptibility, whereas its deletion enhances ACA resistance in *Mycobacterium*.

AcasR directly binds to the promoter region of the fatty acid ligase gene *fadD9* and negatively regulates its expression, establishing this gene as the only functionally validated target gene of AcasR for the first time. In this study, *fadD9* overexpression enhanced mycobacterial resistance to ACA, whereas its deletion increased susceptibility to ACA. These findings suggest that the development of FadD9 inhibitors may potentiate the bactericidal activity of ACA against mycobacteria. 5′-O-[N-(alkanoyl) sulfamoyl] adenosine (alkanoyl adenosine monosulfamate, alkanoyl-AMS) analogs are potential multitarget FadD inhibitors (4).

Transcriptomic analysis revealed that ACA upregulates the expression of genes associated with mycobacterial siderophore biosynthesis and transport, while downregulating that of iron storage and oxidative phosphorylation-related genes, suggesting ACA-induced intracellular iron deficiency. Iron starvation leads to the synthesis and secretion of siderophores (23), downregulates the expression of energy production-related genes (TCA cycle, NADH dehydrogenation, and cellular respiration), and inhibits ribosomal activity (1). LC-MS-based lipidomics further confirmed that ACA stress significantly increases the production of iron-loaded siderophores and SL-1 in mycobacteria. Iron quantification assays demonstrated a significant increase in iron levels in the mycobacterial cell envelope and a marked decrease in cytosolic iron levels in the presence of ACA, indicating enhanced siderophore synthesis under ACA stress. Iron-bound siderophores were not efficiently transported into the cytosol, ultimately inhibiting bacterial growth due to iron deprivation (Figure 8B). ACA targets the CpsA protein in mycobacteria (13). Zhang et al. (34) showed that CpsA1 (Rv3267) plays an important role in iron utilization in mycobacteria, although its precise function remains unclear. In addition to IrtA and IrtB, mycobacteria may possess other transporters involved in siderophore-mediated iron uptake (34). Based on the findings of this and previous studies, we hypothesized that CpsA plays a critical role in siderophore import.

AcasR represses the expression of *fadD9*, leading to downregulation of the expression of siderophore biosynthesis genes and, consequently, reduced siderophore production. Both membrane-associated and cytosolic iron levels were significantly lower in the ACA-treated *fadD9*-knockout strain, likely accounting for the increased susceptibility of the *fadD9* mutant to ACA. This study provides the first evidence linking the fatty acid ligase FadD9 to siderophore biosynthesis. However, the mechanism linking FadD9 to *mbt* repression remains unresolved. The *M. tuberculosis* genome harbors 34 *fadD* genes associated with lipid biosynthesis and metabolism. Mycobacterial *fadD* are grouped into two subclasses based on their functions: fatty acyl-CoA ligases (FACLs), which are involved in lipid degradation, and fatty acyl-AMP ligases (FAALs), which are involved in lipid biosynthesis (3, 16, 26). The precise roles of many FACLs remain poorly characterized, whereas functionally non-redundant FAALs are better understood (4). FadD33 (MbtM) is an essential FAAL that activates lipophilic chains for mycobactin siderophore biosynthesis, thereby enabling mycobacterial iron acquisition from host macrophages (28). While *fadD9* deletion reduces *mbtEDC* transcription and mycobactin levels, it is unclear whether this occurs indirectly via disruption of lipid homeostasis or through a more direct metabolic coupling. One plausible indirect model is that altered fatty acid pools or membrane composition secondarily impacts iron sensing or siderophore biosynthesis, as mycobacterial lipid metabolism and iron acquisition are physiologically interconnected. Alternatively, FadD9 might generate a specific acyl-CoA molecule that directly or via an unknown regulator controls *mbt* transcription, but no evidence supports such direct regulation at present. Distinguishing these possibilities will require further biochemical and metabolomic analyses. Future studies should employ metabolic flux analysis coupled with membrane proteomics to systematically elucidate the molecular mechanisms by which FadD9 regulates mycobactin biosynthesis and transport.

Iron is an essential metal, and *M. tuberculosis* acquires iron from its environment through two different systems: siderophore-mediated iron acquisition (SMIA) and heme-iron acquisition (HIA). These systems involve the uptake and degradation of heme to release ferrous iron (27). MbtB is an essential enzyme that catalyzes the coupling of activated salicylic acid with serine/threonine and the subsequent cyclization to form the phenyloxazoline moiety, which is required for the biosynthesis of salicylate-derived siderophores and mycobacterial growth under iron-limiting conditions (6). In contrast, *ppe37*, an iron-regulated PPE gene, is essential for HIA. However, a nine-amino-acid deletion near the N-terminus of BCG PPE37 (amino acids 31–39 of the *M. tuberculosis* PPE37 protein) underlies the profound defect of BCG in HIA (27). In this study, we constructed the H37Ra/Δ*mbtB* strain, which exhibits growth inhibition under normal conditions. However, partial growth restoration was observed upon supplementation with mycobactin and hemin. When supplemented with 5 μM hemin, the *mbtB*-deficient H37Ra strain showed impaired growth compared to the wild-type strain. However, the *mbtB* mutant grew better than the wild-type strain in the presence of hemin and ACA. This suggests that *Mycobacterium* activates the HIA pathway upon blocking the SMIA pathway. Consequently, ACA, which targets the SMIA pathway, loses its effectiveness, leading to the development of ACA resistance in the bacteria.

Despite these insights, several limitations should be noted. First, our mechanistic studies focused on the transcription factor's role in modulating iron homeostasis and ACA susceptibility, and thus we did not systematically test ACA combined with other clinically relevant anti-TB drugs beyond isoniazid and vancomycin. Given that different modes of iron depletion may differentially affect antibiotic susceptibility, future studies are needed to determine whether ACA-induced iron starvation can enhance the efficacy of a broader range of anti-TB agents, particularly under host-relevant conditions.In summary, this study identified AcasR, a MarR family transcriptional regulator, as a negative regulator of fatty acid ligase gene expression. This protein led to downregulation of the expression of mycobacterial siderophore biosynthesis genes and, consequently, reduced siderophore production. Under ACA treatment, both membrane-associated and soluble iron levels decreased in *fadD9* deletion strain. This mechanism explains the increased ACA susceptibility observed in AcasR-overexpressing strains and *fadD9*-knockout mutants. Our findings suggest a novel strategy for enhancing the bactericidal activity of ACA.

## Data Availability

The datasets presented in this study can be found in online repositories. The names of the repository/repositories and accession number(s) can be found in the article/Supplementary Material.
