# Supplementary material for: The AcasR-FadD9 regulatory axis modulates iron homeostasis and susceptibility to the anti-mycobacterial compound ACA
Source: Front Tuberc. 2026 Jun 30;4:1830528. doi: 10.3389/ftubr.2026.1830528 (PMC13491442; doi:10.3389/ftubr.2026.1830528)
Supplement: Supplementary file 1 [file Datasheet1.docx]

Supplementary Material

**The AcasR-FadD9 regulatory axis modulates** **iron homeostasis and susceptibility to the anti- mycobacterial compound ACA**

**Feng Huang^1^, Zixin Wang^1^, Dong Jiang^1^, Shengyi Li^1^, Zheng-Guo He^2,3^,** **Hua Zhang^1^***

^1^National Key Laboratory of Agricultural Microbiology, College of Life Science and Technology, Huazhong Agricultural University, Wuhan 430070, China

^2^State Key Laboratory of Virology, Taikang Center for Life and Medical Sciences, TaiKang Medical School (School of Basic Medical Sciences), Wuhan University, Wuhan, 430071, China

^3^College of Life Science and Technology, Guangxi University, Nanning 530004, China

*** Correspondence:**
Hua Zhang
[zhanghua@mail.hzau.edu.cn](mailto:zhanghua@mail.hzau.edu.cn)

**List of Supplementary material**

**Supplementary Figure 1** PCR verification of acasR, fadD9 and mbtB knockout strain.

**Supplementary Figure 2** Effects of AcasR and FadD9 on ACA sensitivity or resistance of *M. bovis* BCG.

**Supplementary Figure 3** AcasR orthologs in *M. bovis* BCG, *M. tuberculosis* H37Rv and *M. tuberculosis* H37Ra.

**Supplementary Figure 4** Transcriptomic assays for the effect of ACA and *fadD9* deletion on the gene expression of *Mycobacterium bovis* BCG.

**Supplementary Figure 5** The deletion of the *mbtB* gene contributes to the resistance of H37Ra to ACA.

**Supplementary Table 1** Sequences of PCR primers used in this work

**Supplementary Table 2** Table plasmids used in this work

**Supplementary Table 3** Summary statistics of whole genome sequencing

## Supplementary Figures

**Supplementary Figure 1 PCR verification of *acasR*, *fadD9* and mbtB knockout strain.** PCR amplification was performed using the genome of knock out strain as a template. (A) M: marker; Lane 1, acasR (WT/BCG genome); Lane 2, *hyg* (WT/BCG genome); Lane 3, *acasR* (*acasR*-deleted BCG genome); Lane 4, *hyg* (*acasR*-deleted BCG genome); Lane 5, *fadD9-*NTD (WT/BCG genome); Lane 6, *fadD9-*CTD (WT/BCG genome); Lane 7, *hyg* (WT/BCG genome); Lane 8, *fadD9-*NTD (*fadD9*-deleted BCG genome); Lane 9, *fadD9-*CTD (*fadD9*-deleted BCG genome); Lane 10, *hyg* (*fadD9*-deleted BCG genome). The lengths of genes were shown as follows: *acasR*, 432bp; *hyg*, 999 bp; *fadD9-NTD*, 1935bp; *fadD9-CTD*, 1254bp. (B) M: Marker; Lane 1: *mbtB* (374bp-1179bp, 806bp), WT/H37Ra genome; Lane 2: *hyg* (999bp), WT/H37Ra genome; Lane 3: *mbtB*, (374bp-1179bp, 806bp), *mbtB* -deleted H37Ra genome; Lane 4: *hyg* (999bp), *mbtB*-deleted H37Ra genome.

**Supplementary Figure 2 Effects of AcasR and FadD9 on ACA** **sensitivity or resistance of *M. bovis* BCG.** (A) *acasR* gene attributes mycobacterial sensitivity to ACA. Wild type *M. bovis* BCG (BCG/pMind), *acasR*-deleted strain (△*acasR*/pMind), complemented strain (△*acasR*/pMind-P*_acasR_*-*acasR*) and *acasR*-overexpressing strain (BCG/pMind-*acasR*) were 10×diluted to different concentrations, and equal amounts of culture were spotted on 7H10 plates with (right panel) or without 12 μg/ml ACA (left panel). (B) Freshly grown strains of wild type *M. bovis* BCG (BCG/pMV261), *acasR*-deleted strain (△*acasR*/pMV261) and *fadD9*-overexpressing strain (BCG/pMV261-*fadD9*) were 10× diluted to different concentrations, and equal amounts of culture were spotted on 7H10 plates with (right panel) or without 12 μg/ml ACA (left panel).

**Supplementary Figure 3 AcasR orthologs in *M. bovis* BCG, *M. tuberculosis* H37Rv and *M. tuberculosis* H37Ra.**

**Supplementary Figure 4 Transcriptomic assays for the effect of ACA and *fadD9* deletion on the gene expression of *Mycobacterium bovis* BCG.** (A) The volcanic diagram of gene expression difference between the control samples (wild-type *M. bovis* BCG) and the samples treated with ACA (15 μg/ml) for 26 h determined by RNA Sequencing assays. The significantly upregulated genes were shown by red spots. Downregulated and not significant change genes were shown by blue and gray spots, respectively. (B) The volcanic diagram of gene expression difference between wild-type *M. bovis* BCG and *fadD9*-deleted *M. bovis* BCG strains determined by RNA Sequencing assays. The significantly upregulated genes were shown by red spots. Downregulated and not significant change genes were shown by blue and gray spots, respectively.

**Supplementary Figure 5 The deletion of the *mbtB* gene contributes to the resistance of H37Ra to ACA. (A)** Wild type *M. tuberculosis* H37Ra (H37Ra-WT) and *mbtB*-deleted strain (H37Ra△*mbtB*) were 10×diluted to different concentrations, and equal amounts of culture were spotted on 7H10 plates without any other supplement or with 40 ng/ml mycobactin or 5 μM hemin or 5 μM hemin and 12 μg/ml ACA. **(B)** Wild type *M. tuberculosis* H37Ra (H37Ra-WT) and *mbtB*-deleted strain (H37Ra△*mbtB*) were grown in 7H9 medium without any drug or with 5 μM hemin **(C)** or with 5 μM hemin and 12 μg/ml ACA **(D)**. The concentration of supplement was shown in the figure. At indicated time points, samples were taken and CFU counts were performed. All error bars in the figures represent the standard deviations (SD) of the data derived from three biological replicates. Asterisks (*) denote a significant difference between the recombinant strain and wildtype strain (**, P<0.01; ***, P<0.001). The P-values were calculated by unpaired two-tailed Student’s t-test using GraphPad Prism 8.

**Supplementary Table1 Sequences of PCR primers used in this work**

| **Gene** | **Forward (5’→3’)** | **Reverse (5’→3’)** |
| --- | --- | --- |
| *acasR* | GTACGAATTCATGTGCTTGACAGCGATG  CGCG | TGCTGATCTAGATCACGGGCTTTCGTCGACCA |
| *fadD9* | GCGCCATATGATGTCGATCAACGATCAGCG, | ATATGGATCCTCACAGCAGCCCGAGCAGTC |
| *acasR* upstream | GGTCTTAATTAAGCGTTCAGCCAACTCAGATA | ATATACTAGTCACAGCTGAGGTTACAACGGAA |
| *acasR* downstream | GGCTAAGCTTGTGACCGAAGGCCGTTGT | ATATGCTAGCGAGACCGACGGCCAACAG |
| *fadD9* upstream | GGCCTTAATTAAAGAGCGGCTTCGGTCCGTTC | ATATACTAGTCACGCGGTCACCTCGGCGTGCC |
| *fadD9* downstream | ATGAAAGCTTCGGGTAACCAAGCAGCCCGT | ATATGCTAGCCAAGGAATAGACCGCGGCCA |
| *fadD9-CTD* | GCGCCATATGATGTCGATCAACGATCAGCG | AGACTCTAGATCACGCATCGGGACCGCTTT |
| *hyg* | ATATCATATGATGGACGCTGCCACCCTGGC | ATGATCTAGATCACAGCAGCCCGAGCAGTC |
| *acasR*p | ATATGAATTCGTTGCCAGCCCGGCCACTGT | GCGCTCTAGAAGCTGAGGTTACAACGGAAG |
| *fadD9*p | AGACGAATTCTCCGGAACCACCGAGCCCGA | ATATTCTAGAGCGGTCACCTCGGCGTGCCG |
| *Rv1219*p | GGCTCACTCCGGCACTGGTG | AATCCGTGCCGACCGAACTG |
| *mbtB* upstream | ATATTAATTAACAGCACCACCAGATAGACGT | ATATAACTAGTCACAGCGACATCATCCGGAT |
| *mbtB*  down stream | AGCGAAGCTTCAGCGGCTATTCATGTCC | ATATGCTAGCCGCACCTCGGCGAGGACA |
| *mbtB* | AATATGCGGCCGCAGTGGTGCATGCTACGGCGT | CGCCCCCCTCTAGATTATCGGACATCGGCACTC |

**Supplementary Table 2 Table plasmids used in this work**

| Plasmid Relevant features Source | | |
| --- | --- | --- |
| pMind-*acasR* | *acasR* gene cloned in pMind | This work |
| pMind-P*_acasR_*-*acasR* | *acasR* gene with its promoter cloned in pMind | This work |
| pMind-*acasR*up-*acasR*dn-*lacZ* | *acasR* gene upstream 972 bp and downstream 1004 bp cloned in pMind-*lacZ* | This work |
| pET-*acasR* | *acasR* gene cloned in pET | This work |
| pMV261-*lacZ* | *lacZ* gene cloned in pMV261 | This work |
| pMV261-*fadD9*p-*lacZ* | *fadD9* promoter cloned in pMV261-*lacZ* | This work |
| pMV261-*acasR*p-*lacZ* | *acasR* promoter cloned in pMV261-*lacZ* | This work |
| pMV261-*fadD9* | *fadD9* gene cloned in pMV261 | This work |
| pMind-*fadD9*up-*fadD9*dn-*lacZ* | *fadD9* gene upstream 1000bp and downstream 998bp cloned in pMind-*lacZ* | This work |
| pMind-*mtbB*up-*mbtB*dn-LacZ | *mtbB* gene upstream fragment (up 857bp+ ORF134bp) and downstream fragment (ORF214bp+dn 797bp) cloned in pMind-*lacZ* | This work |

**Supplementary Table 3 Summary statistics of whole genome sequencing**

| Sample Name | Length | Covered Base | Mapped Base | Mapped Reads | Coverage (%) | Depth |
| --- | --- | --- | --- | --- | --- | --- |
| BCG_R01 | 4,374,522 | 4,364,471 | 6,377,326,567 | 53,490,229 | 99.77 | 1457.83× |
| BCG_WT | 4,374,522 | 4,364,518 | 7,805,638,754 | 81,625,955 | 99.77 | 1784.34× |
